# Temporal Fluctuations of Suicide Mortality in Japan from 2009 to 2023 Using Government Databases

**DOI:** 10.3390/ejihpe14040071

**Published:** 2024-04-21

**Authors:** Ryusuke Matsumoto, Eishi Motomura, Motohiro Okada

**Affiliations:** Department of Neuropsychiatry, Division of Neuroscience, Graduate School of Medicine, Mie University, Tsu 514-8507, Japan; matsumoto-r@clin.medic.mie-u.ac.jp (R.M.); motomura@clin.medic.mie-u.ac.jp (E.M.)

**Keywords:** suicide mortality, COVID-19, Japan, gender

## Abstract

In Japan, suicide mortalities consistently decreased before the COVID-19 pandemic (from 2009 to 2019) but, conversely, increased after the pandemic outbreak from 2020 to 2022. To provide up-to-date suicide statistics in Japan, this study determined the temporal fluctuations of standardized suicide mortalities (SMRs), disaggregated by sex and age, by joinpoint regression analysis using the government suicide database, named the “Basic Data on Suicide in Region”. From January 2009 to December 2023, three temporal fluctuation patterns of SMRs pertaining to working age and older adults were detected, such as attenuations of decreasing trends before the COVID-19 pandemic (from around the mid-2010s), a sharply increasing trend that coincided with the pandemic outbreak, and gradually decreased during the pandemic, but no changes at the end of the COVID-19 pandemic. In particular, the SMRs of working-age females sharply increased concurrently with the pandemic outbreak, whereas those of males did not change. However, before the pandemic, decreasing trends of the SMRs of working-age males diminished in the mid-2010s, but those of females consistently decreased. The SMRs of working-age males indicated non-significant but sharply increasing trends in early 2022, a trend that was not observed for females. In contrast to working-age adults, the SMRs of adolescents already began to increase in the mid-2010s and also indicated consistently increasing trends between the periods during and after the pandemic. These results suggest, contrary to our expectations, that the impacts of both the outbreak and end of the COVID-19 pandemic were limited regarding the increase in SMRs from 2020. Therefore, when revising suicide prevention programs in the post-COVID-19 era, it should be noted that focusing on pandemic-associated factors alone is not sufficient.

## 1. Introduction

From 2009 to 2019, suicide mortality in Japan consistently decreased [1,2,3,4,5,6,7]. The World Health Organization (WHO) developed the “Comprehensive Mental Health Action Plan” with the goal of reducing the global suicide mortality rate by 10% from 2012 to 2020 [8]. Japan successfully achieved a 20% reduction in standardized suicide mortality rates (SMRs) per 100,000 population, decreasing from 21.99 (2012) to 16.58 (2020) [9,10,11,12,13]; however, several time series analyses reported that SMRs in Japan began increasing from 2020 [14,15,16,17]. These reports speculated that the recent increase in SMRs in Japan might be caused by factors associated with the COVID-19 pandemic, since this increase was observed to coincide with the COVID-19 pandemic outbreak [18,19,20,21,22,23,24,25]. 

In the early phase of the COVID-19 pandemic, various reports concerned the possibility that suicides would increase due to socioeconomic and/or psychosocial deteriorations induced by the COVID-19 pandemic itself or several restriction measures for the prevention of spreading the COVID-19 [26,27,28]. Many findings have been considered to reflect these concerns, and, as such, increasing SMRs in Japan from 2020 have been accepted as a phenomenon caused by the COVID-19 pandemic. However, to date, the majority of studies have reported that suicide rates decreased or remained unchanged in major Organization for Economic Co-operation and Development (OECD) countries during the pandemic, except for Japan, South Korea and Spain [16,17,29,30,31,32,33,34,35].

It has been reported that the impacts of the COVID-19 pandemic were not uniform, with specific vulnerable groups being uniquely affected [14,15,36,37,38,39]. In particular, lifestyle changes, including opportunities for education, coping with stress and the lack of recreation opportunities caused by social restriction measures and the rise in unemployment induced by economic deterioration could have contributed to increasing suicidal risks for some vulnerable individuals, such as the elderly/younger generation, psychiatric patients, unemployed individuals and healthcare workers [36,37,38]. The high-risk groups for suicides during the COVID-19 pandemic in Japan have been identified to be males <30 and females <60 years [14,15,16,17,23,40,41]. In particular, temporal fluctuations in the SMRs of these high-risk groups sharply increased with the COVID-19 pandemic outbreak, and SMRs increased up to 2021 [14,15,38]. Similar to Japan, the increasing suicide rates of working-age females during the initial stage of the COVID-19 pandemic were also reported in Spain and South Korea [33,34,35]. The actual mechanisms underlying the increasing SMRs of high-risk groups in Japan remain to be clarified; however, a number of reports concluded that this increase during the COVID-19 pandemic involved some factors associated with the pandemic itself, such as socioeconomic or psychosocial deteriorations [13,19,20,21,22,42,43,44,45].

In May 2023, the WHO and Japanese government declared the end of the COVID-19 pandemic [46,47]. Analyzing fluctuations in SMRs, disaggregated by age and sex, can provide important findings to help plan suicide prevention programs after the COVID-19 pandemic era. The actual causes of increasing SMRs from 2020 in Japan remain to be clarified. Although the actual causes of increasing SMRs from 2020 in Japan remained to be clarified, where SMRs recovered after the end of the COVID-19 pandemic, we should explore the specific factors that exacerbated and improved respective during and after the pandemic, for evidence-based planning for suicide prevention programs for the post-COVID-19 pandemic era. Conversely, where the high levels of SMRs during the pandemic persisted after the end of the pandemic, the recent increasing SMRs in Japan might be induced by the other factors that coincidentally developed with the pandemic outbreak. Furthermore, we should also pay attention to the possibility that the suicidal risk which developed with the COVID-19 pandemic outbreak could not be improved after the end of the pandemic. Based on these possibilities, in order to clarify the fluctuations of SMRs disaggregated by age and sex before, during and after the COVID-19 pandemic, the present study determined the temporal fluctuations of SMRs disaggregated by sex and age from January 2009 to December 2023 in Japan using joinpoint regression analysis.

## 2. Materials and Methods

### 2.1. Data Sources

The Japanese government has two national suicide databases, the “Vital Statistics Registration” (VSR), collected by the Ministry of Health, Labor and Welfare (MHLW) [9,48,49], and the “Suicide Statistics” (SSNPA), collected by the National Police Agency (NPA) [50]. The VSR publishes a complete coverage of all Japanese deaths that have occurred in Japan, with the cause of death coded by ICD-10, and it has currently published suicide statistics up to 2019. In Japan, only medical doctors can prepare death certificates, and the Medical Practitioners Law stipulates that abnormal deaths, including probable suicides, must be reported to the NPA within 24 h. The NPA must examine all corpses with abnormal causes of death to determine the cause of death by conducting physiological examinations [50]. The SSNPA provides data on the number of individuals who have died by suicide in each region under the jurisdiction of local police stations. The judicial police investigate the personal characteristics and background factors of each suicide case [50]. The MHLW provides the “Basic Data on Suicide in Region” (BDSR) for public access, which is a compilation of SSNPA data organized into detailed categories, such as sex, age, nationality and dwelling place [51]. The monthly suicide numbers from January 2009 to December 2023, disaggregated by sex (males and females) and age (<20, 20–29, 30–39, 40–49, 50–59, 60–69, 70–79 and >80 years old), were obtained from the BDSR provided by the MHLW [51]. Populations disaggregated by sex and age were obtained from the “Surveys of Population, Population Change and the Number of Households based on the Basic Resident Registration” published in e-Stat (Ministry of Internal Affairs and Communications) [52]. 

Monthly SMRs disaggregated by sex and age were calculated by dividing the monthly suicide numbers by the population of the corresponding groups in the same year [40]. Finally, the monthly SMRs were converted to annualized values for 365 days.

### 2.2. Statistical Analyses 

Joinpoint regression and interrupted time series analyses are well-established time series statistical methods used for analyzing temporal fluctuations in SMRs. As has been noted, suicide is a temporally and fundamentally complicated phenomenon comprising various risk factors [16,20,23,32,40,41,53]. Interrupted time series analysis is known as one of the most effective/powerful statistical methods for detecting the impacts of the COVID-19 pandemic outbreak on SMRs via the correlation between the periods before and after the pandemic outbreak [13,22,54,55,56]. Interrupted time series analysis can incorporate various options, including parametric/non-parametric regressions, seasonal variation and panel data analyses [15,54,55,56], but it cannot detect unknown joinpoints (changing trends periods) during observation periods. Indeed, previous reports have suggested, when the intervention is set at the COVID-19 pandemic outbreak alone, interrupted time series analysis tends to overestimate the positive impacts of the pandemic outbreak on SMRs due to the attenuation of decreasing trends of male SMRs before the pandemic (in the late 2010s) [15,23,39,41]. In contrast, joinpoint regression analysis has been evaluated to be an appropriate statistical method, which can detect unknown joinpoints, where trends change via fitting the simplest joinpoint model that the trend data allow [57,58]. Based on these statistical backgrounds, to analyze the temporal fluctuations in SMRs in Japan from January 2009 to December 2023, this study adopted Joinpoint Regression Program ver4.9.1.0 (the National Cancer Institute, Bethesda, Maryland) [16,17,40,58]. A detailed description of the methods used in the Joinpoint Regression Software is given in the user manual published by the National Cancer Institute (NCI). 

### 2.3. Ethics

The funding source of this study helped to define the research questions and assisted with data interpretation, but it had no role in the model development, parameterization or the methodological aspects of the study. Although the Medical Ethics Review Committee of Mie University waived the need for ethical approval due to the use of publicly available governmental data, this study adhered to the Strengthening the Reporting of Observational Studies in Epidemiology (STROBE) guidelines. There are no missing data in this study.

## 3. Results

### 3.1. Fluctuation in SMRs from 2009 to 2023

**Figure 1:**
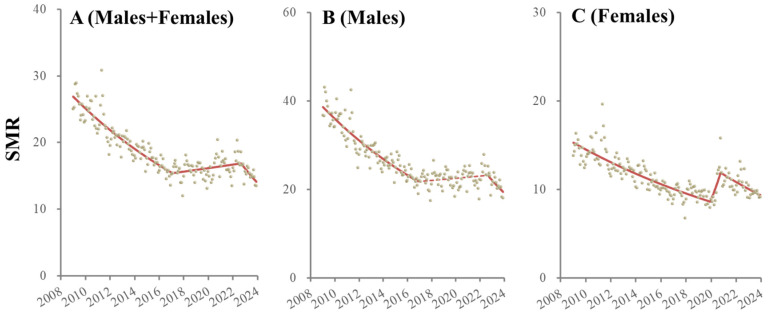
Temporal fluctuation of SMRs of males + females (**A**), males (**B**) and females (**C**) from January 2009 to December 2023 in Japan analyzed using joinpoint regression analysis. Ordinate and abscissa indicate the annualized monthly SMRs (per 100,000 population) and years, respectively. Grey circles indicate the observed monthly SMR values. Red lines indicate the results calculated by joinpoint regression analysis. Solid and dotted red lines indicate the significant and non-significant trends of SMRs, respectively.

Two joinpoints were detected in the temporal fluctuations in the SMRs of males + females between January 2009 and December 2023. The SMRs of males + females decreased significantly from January 2009 to January 2017, but this reversed from February 2017 to September 2022. From October 2022 to December 2023, the SMRs of males + females began to significantly decrease again (Figure 1 and Table 1). 

Two joinpoints of male SMRs during the observation period were also detected. Male SMRs decreased from January 2009 to December 2016 but did not significantly change from January 2017 to September 2022. Similar to the SMRs of males + females, after October 2022 to December 2023, male SMRs significantly decreased (Figure 1 and Table 1). 

Two joinpoints of female SMRs that were, critically, dissimilar to those of males, were also detected. Female SMRs decreased from January 2009 to December 2019, but they significantly and sharply increased from January 2020 to October 2020 and significantly decreased from November 2020 to December 2023 (Figure 1 and Table 1). 

### 3.2. Fluctuations in Male SMRs Disaggregated by Age from 2009 to 2023

**Figure 2:**
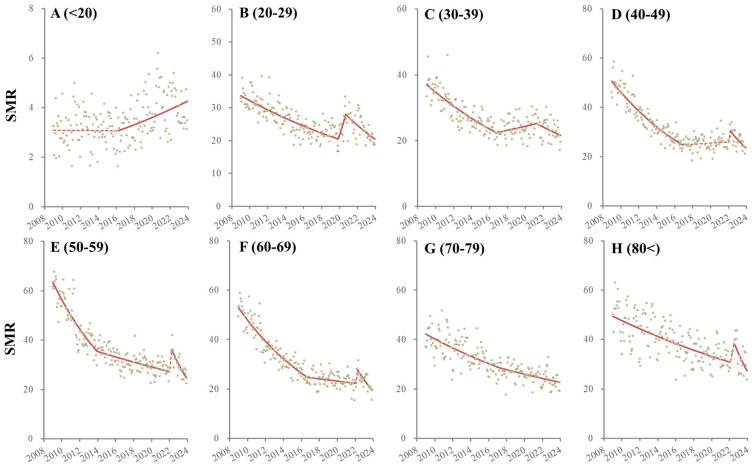
Temporal fluctuation of males SMRs disaggregated by age, such as younger than 20 (<20: (**A**)), 20–29 (**B**), 30–39 (**C**), 40–49 (**D**), 50–59 (**E**), 60–69 (**F**), 70–79 (**G**) and over 80 years (80<: (**H**)) from January 2009 to December 2023 in Japan analyzed using joinpoint regression analysis. Ordinate and abscissa indicate the annualized monthly SMRs (per 100,000 population) and years, respectively. Grey circles indicate the observed monthly SMR values. Red lines indicate the results calculated by joinpoint regression analysis. Solid and dotted red lines indicate the significant and non-significant trends of SMRs, respectively.

The male SMR for those aged <20 did not change until March 2016, but after April 2016, it consistently increased (Figure 2 and Table 2). The SMR for males aged 20–29 decreased from January 2009 to November 2019, but it significantly and sharply increased from December 2019 to September 2020 and significantly decreased from October 2020 to December 2023 (Figure 2 and Table 2). The SMR for males aged 30–39 decreased from January 2009 to October 2016 but reversed to significantly increase from November 2016 to April 2021 and then significantly decreased from May 2021 to December 2023 (Figure 2 and Table 2). The SMR for males aged 40–49 decreased from January 2009 to November 2016. It did not significantly change from December 2016 to December 2021 but non-significantly sharply increased (from January 2022 to March 2022) and significantly decreased from April 2022 to December 2023 (Figure 2 and Table 2). The SMR for males aged 50–59 consistently decreased from January 2009 to November 2013, but the decreasing trends attenuated from December 2013. Non-significant but sharply increasing trends from January 2022 to April 2022 were observed followed by a significant decrease from May 2022 to December 2023 (Figure 2 and Table 2). The SMR for males aged 60–69 also consistently decreased from January 2009 to January 2022, but the decreasing trends attenuated from October 2016. A non-significant but sharp increase from February 2022 to April 2022 was also observed, followed by a significantly decreasing trend from May 2022 to December 2023 (Figure 2 and Table 2). The SMR for males aged 70–79 consistently decreased during the observation period, but the decreasing trends attenuated from March 2017 (Figure 2 and Table 2). The SMR for males over 80 consistently decreased from January 2009 to January 2022. A non-significant but sharp increase from February 2022 to July 2022 was observed, followed by a significantly decreasing trend from August 2022 to December 2023 (Figure 2 and Table 2). 

### 3.3. Fluctuations in Female SMRs Disaggregated by Age from 2009 to 2023

**Figure 3:**
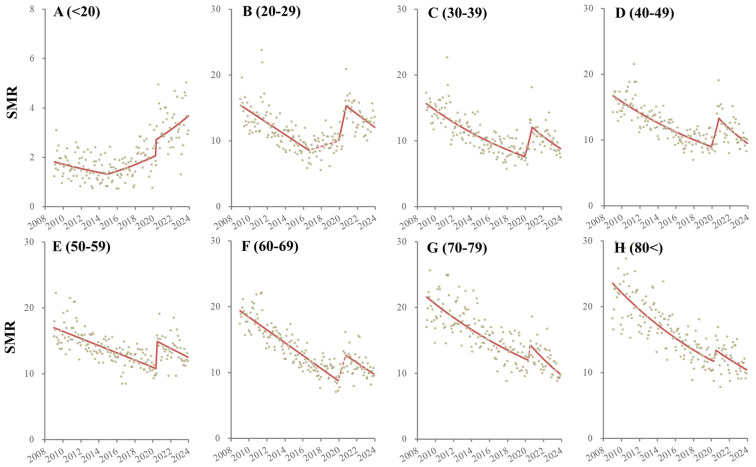
Temporal fluctuation of male SMRs disaggregated by age, such as younger than 20 (<20: (**A**)), 20–29 (**B**), 30–39 (**C**), 40–49 (**D**), 50–59 (**E**), 60–69 (**F**), 70–79 (**G**) and over 80 years (80<: (**H**)) from January 2009 to December 2023 in Japan analyzed using joinpoint regression analysis. Ordinate and abscissa indicate the annualized monthly SMRs (per 100,000 population) and years, respectively. Grey circles indicate the observed monthly SMR values. Red lines indicate the results calculated by joinpoint regression analysis. Solid and dotted red lines indicate the significant and non-significant trends of SMRs, respectively.

The SMR for females aged < 20 decreased from January 2009 to December 2014, but after January 2015, it increased. In particular, the SMR for females aged < 20 sharply increased with the COVID-19 pandemic outbreak (Figure 3 and Table 2). 

The SMR for females aged 20–29 decreased from January 2009 to August 2016, but this changed, non-significantly increasing from September 2016 to December 2019. However, the period January 2020 to November 2020 indicated a significant and sharp increase in the SMR followed by a significantly decreasing trend from December 2020 to December 2023 (Figure 3 and Table 2). The SMR for females aged 30–49 consistently decreased from January 2009 to December 2019, but it significantly and sharply increased with the pandemic outbreak (from January 2020 to October 2020), which was followed by a significant decrease from November 2020 to December 2023 (Figure 3 and Table 2). The SMRs of females aged 50–59 consistently decreased from January 2009 to April 2020 but significantly and sharply increased with the pandemic outbreak (from May 2020 to August 2020). This was followed by a significantly decreasing trend from September 2020 to December 2023 (Figure 3 and Table 2). The SMRs of females aged > 60 also indicated consistently decreasing trends during the observation period, whereas non-significant but sharply increasing trends were observed with the pandemic outbreak (Figure 3 and Table 2).

## 4. Discussion

Using joinpoint regression analysis, this study revealed the age- and sex-dependent temporal fluctuation patterns of suicide mortalities from January 2009 to December 2023 in Japan. In general, the SMRs of males aged 20–29 and females aged 30–59 consistently decreased before the COVID-19 pandemic outbreak, but they sharply increased with the outbreak and decreased between the period during and after the COVID-19 pandemic. Notably, we did not observe a change in the SMRs of these high-risk groups that coincided with the end of the COVID-19 pandemic. The SMRs of females over 60 years of age also consistently decreased before the COVID-19 pandemic outbreak but non-significantly and sharply increased with the pandemic outbreak; this was followed by a decrease between the period during and after the COVID-19 pandemic. On the contrary, the decreasing trends of SMRs of working-age (30–69) males attenuated from the mid-2010s. The SMRs of males aged 40–69 did not indicate changes with the COVID-19 pandemic outbreak, but they non-significantly and sharply increased in early 2022 and decreased between the period during and after the pandemic. The trends of SMRs of males and females < 20 began to increase before the pandemic outbreak (in mid 2010s); however, the SMR of females < 20 sharply increased with the COVID-19 pandemic outbreak, whereas the SMR of males < 20 did not respond to the pandemic outbreak. Contrary to our expectations, we did not detect any decreasing SMRs with the end of the pandemic. Therefore, the temporal fluctuation patterns of the SMRs of males younger than 30 and all females were similar to the results of previous reports that analyzed fluctuations from 2009 to 2022 using joinpoint regression analysis [14,15,39]; however, the non-significantly but sharply (transiently) increasing SMRs of working-age males in early 2022 possibly contributed to the tendency of previous reports to underestimate the decrease in SMRs in late 2022 [14,15,39]. 

### 4.1. Suicides among Adolescents and the Elderly

Between 2009 and 2021, suicide was the leading cause of death among adolescents in Japan [59,60]. Although suicide rates among adolescents have globally been increasing over time, most studies from other OECD countries have reported that adolescent SMRs decreased or remained unchanged during the pandemic [61,62,63,64,65], whereas in Japan, it has been reported that the SMRs of both males and females under 20 years old increased during the pandemic [39,40,41,66,67]. These reports attributed the increase in adolescent SMRs to major lifestyle changes during the pandemic, particularly pertaining to the impaired ability to engage in recreational and educational opportunities and being forced to spend much of their time at home due to social restriction measures [39,40,41,66,67]. In contrast, several reports have suggested that the adolescent SMRs of both males and females in Japan had already begun to increase before the pandemic outbreak [16,17,23,40,41]. This study not only substantiated that the SMRs of the generation aged < 20 had begun to increase before the pandemic but also identified that the COVID-19 pandemic outbreak was probably not a primary cause for increasing SMRs for those aged < 20.

Adolescence is a period of psychosocial and biological development that involves various social stages (e.g., middle school, high school, university, special vocational school and work) [68]. A recent study, focusing on evidence-based policy making, suggested that recently increasing adolescent SMRs in Japan may be the result of national suicide prevention programs for young populations in the “General Principles of Suicide Prevention Policy”, which place too much emphasis on making improvements in schools and relatively less on improving home environments, which play a key role in child rearing [39,41]. The primary causes of student SMRs were found to be worrying about the future and underachievement (in school-related problems) and mental illness (depression and anxiety disorders) [40,41]. Considering that the onset of internalizing disorders is at approximately 15 years of age, internalizing symptoms have likely played important roles in the recently increasing SMRs among adolescents [40,41,69,70]. Indeed, the prevalence of internalizing disorders among adolescents has been increasing in Japan [71]. Although it may be excessive over discussion, the decreasing birth rates may contribute to increasing internalizing symptoms [39,41]. Progressively decreasing birth rates have continuously increased for children without siblings, with rates of >20% in 1997 and >30% in 2015 [72,73]. 

In the past, emergence of second child/siblings had been argued to contribute to developmental crisis for firstborn children by earlier psychodynamics [74,75]; however, firstborn children with siblings display positive or regular responses, appear to quickly adapt to new tasks and express more positive emotions and fewer separation reactions and dependent behaviors in comparison to only children [76,77,78,79,80,81,82]. Additionally, parents tend to adopt strategies of high care and/or high control when raising an only child [40,41,74,75]. It has been established that low-care parenting attitudes negatively affect developmental models regarding the competent/worthy self-model and reliable/supportive relationships with others, with persistence to adolescence, resulting in increasing risks of internalizing disorders and suicide [83,84,85,86,87,88,89,90,91,92]. However, excessive high-control parenting also plays an important role in the depressive mood and feelings of hopelessness among students [93]. Furthermore, mothers with internalizing symptoms/disorders tend to adopt affectionless control styles (low care with high control) [93]. These psychological findings suggest the possibility that maternal internalizing symptoms/disorders and high-control parenting may induce a vicious negative cycle that increases the prevalence of internalizing disorders in later generations [41].

Aging is not only biological, but also a social/cultural phenomenon affected by ethnicity, class, gender and the political and economic climate [94]. According to the stages of psychosocial development, elderly is placed in the last stage, that of ego integrity or despair [95]. Globally, the SMRs of the elderly are higher than those of the younger generation [96]. Despair, loneliness and the death of a meaningful person or spouse may be suicide risk factors among the elderly [97]. Regarding the elderly living alone, possibilities of both economic poverty and healthcare vulnerability emerge [35]. However, it is noteworthy that SMRs among the elderly have not increased in some countries, such as Spain, South Korea and Japan, where SMRs among other age groups increased during the COVID-19 pandemic [16,17,33,34,35]. The findings regarding the consistently decreasing SMR trends of the elderly in Japan before, during and after the COVID-19 pandemic suggest that the impacts of changes in community/society environments for the elderly might be smaller than expected.

### 4.2. Suicide in Working-Age Females

Various studies have revealed that working-age females were a high-risk group for suicide during the pandemic [13,15,16,17,18,19,21,22,23,25,40,41,42,43,44,45]. This study also demonstrated that female SMRs for those under 60 sharply increased with the COVID-19 pandemic outbreak, whereas the ending of the pandemic did not affect them. On the contrary, the fluctuation in female SMRs for those over 60 years old related to neither the outbreak nor the ending of the pandemic. Furthermore, other than one sharp increase synchronized with the pandemic outbreak, the trends of female SMRs for those over 30 years old consistently decreased overall in the observation period. These common temporal fluctuations in female SMRs for those over 30 suggest that some kind of turmoil in the initial stage of the COVID-19 pandemic, rather than the pandemic itself, may have contributed to the increase in the SMRs of females over 30.

It is well known that the overall unemployment rates in Japan drastically increased with the COVID-19 pandemic outbreak, which was followed by a recovery [15,16,17]. Based on the similarity between the SMRs of working-age females and the overall unemployment rates in Japan during the pandemic, several studies have found links between them [15,16,17]. Before the pandemic outbreak, the positive fixed effects of unemployment rates on female SMRs could be detected, whereas this relation was abolished during the pandemic [16,17]. Unexpectedly, the increasing rates of short-term unemployment (shorter than three months of unemployment) contributed to increasing female SMRs, but relatively longer-term unemployment did not [15,16,17]. Traditionally, unemployment due to recessions has been established as a major risk for suicide; however, the impact of unemployment on male suicide has received more attention [98,99,100,101,102]. It is well known that SMRs showed a drastic increase during the Asian financial crisis, and female SMRs also increased by approximately 20%, from 12.4 (1997) to 15.3 (1998) [11,100,101,102,103]. In this study, it was shown that female SMRs in 2020 (10.74) increased by approximately 10% compared to 2019 (9.27). Therefore, the impacts of increasing shorter-term unemployment among females was at least partially involved in the sharp increase in the SMRs of working-age females.

### 4.3. Suicide in Working-Age Males

The temporal fluctuations in the SMRs of working-age males indicated quite different patterns to those of females, since a sharp increase in the SMRs of working-age males coinciding with the COVID-19 pandemic outbreak was not observed. However, in early 2022, the SMRs of males aged 40–69 non-statistically but sharply increased. Despite the statistical characterization of joinpoint regression analysis, which detects the transformation of trends through fitting the simplest joinpoint model, it could not detect the transiently increasing trends in early 2022 as a significant change. Therefore, considering the causes for transient increases in the SMRs of working-age males may provide important insights into exploring the mechanisms of suicide among working-age males.

The transiently increasing trend in early 2022, while not statistically significant, was a common phenomenon among the SMRs of working-age males. Previous context can provide two potential causes underlying this sharp increase. The first is an increase in unemployment rates with specific features that selectively affected the SMRs of working-age males. The fluctuations in the total overall unemployment rate indicated consistently and linearly decreasing trends before the COVID-19 pandemic, such as from 5.5% to 2.5% in 2010–2019, whereas unemployment rates sharply increased concurrently with the pandemic outbreak to >3.0% and gradually decreased during the pandemic [15,104]. However, the peak of increasing unemployment rates for over 12 months lagged to 2021 [104]. Unemployment rates for over 12 months specifically contributed to increasing SMRs of working-age males, and their positive impacts continue over 1 year, whereas unemployment rates shorter than 6 months did not significantly relate to male SMRs [15]. Therefore, the temporal positive impacts of unemployment rates lasting over 12 months on the SMRs of working-age males can provide a plausible explanation for the causes of the transient increases in the SMRs of working-age males in early 2022.

Moreover, the revision of various government supportive countermeasures against the economic deterioration caused by COVID-19 in December 2021 [105] may have been a possible factor for the transiently increasing SMRs of working-age males, which was followed by an immediate recovery in early 2022. The government’s supportive countermeasures against economic deterioration caused by COVID-19 were composed of two financial support systems categorized for enterprises and individuals. In December 2021, the government’s supportive countermeasures for enterprises, such as the “Sustainability Benefit”, were revised to the “Business Revitalization Support Fund”, whereas the government implementation of support for individuals related to COVID-19 was continued until May 2023 in the form of “COVID-19 leave support payments and subsidies” [105]. During the COVID-19 pandemic, the governmental welfare and economic support measures had to be revised due to the various changing situations caused by COVID-19. The government’s supportive countermeasures for enterprises were evaluated to be consistently effective during the COVID-19 pandemic, since the number of bankruptcies during the pandemic did not increase compared to before the COVID-19 pandemic [106]. Therefore, it is possible to deny that the revision of government supportive countermeasures for enterprises in December 2021 led to subsequent socioeconomic deterioration. Indeed, transiently and sharply increasing SMRs of working-age males in the early 2022 were immediately recovered.

Recently, it has been suggested that some vulnerable individual groups might have perceived the rapidly changing socioeconomic and political changes during the pandemic as anomic shock [107,108,109,110], contributing to a sharp and transient increase in suicides, which deviated from previous trends [39]. Durkheim conceptualized the anomic suicide theory; namely, that in both economic recessions and booms, social systems are unable to sufficiently adapt to individual needs, leading to increasing suicides via weakened social integration [108,109,110]. In reality, the outbreak and end of the COVID-19 pandemic and/or social restriction measures had heterogeneous psychological effects [111]. Early in the pandemic, individuals suffered from stress due to the forced drastic changes in lifestyle and social systems. Indeed, the increasing SMRs of males aged 20–29 and females < 60 years of age sharply increased after the pandemic outbreak [14,15,16]. The revision of government economic supportive measures from providing compensation for social restriction measures to supporting economic resumption, implemented in late 2022, might be interpreted/perceived to have forced individuals to adapt to the “new normal” in the post-COVID-19 pandemic era, manifesting as anomic shock.

Providing an update regarding the fluctuation in suicide mortality in Japan, this study analyzed temporal fluctuations in SMRs disaggregated by sex and age, but it could not analyze any causalities regarding the detected fluctuations due to the delayed publication of several independent variables necessary to carry this out.

## 5. Conclusions

This study demonstrated that the responses of SMRs in Japan to the onset and end of the COVID-19 pandemic, disaggregated by age and sex, were completely inconsistent. The SMRs of females under 60 years old sharply increased with the pandemic outbreak, whereas any changes in the SMRs of high-risk groups at the end of the pandemic could not be detected. Contrary to females, none of the male SMRs (other than the SMRs of males aged 20–29) indicated any changes in fluctuations that correlated with the COVID-19 pandemic outbreak. Additionally, no changes in male SMRs related to the end of the COVID-19 pandemic could be observed. However, the SMRs of working-age (40–69) males non-significantly but sharply increased with the revision of the government’s supportive countermeasures that were implemented to mitigate the economic deterioration caused by COVID-19 (in early 2022). Therefore, the impact of the COVID-19 pandemic on the increase in suicides in Japan since 2020 has been limited. When planning suicide prevention programs after the post-COVID-19 era, in response to the recent increase in suicides in Japan, it should be noted that focusing on pandemic-associated factors alone is not sufficient.

## Figures and Tables

**Table 1:** Summary of temporal fluctuation of SMRs of males + females, males and females.

	Lower Endpoint	Upper Endpoint	MPC	*p*	
Male + Female	2009/1	2017/1	−0.57	<0.01	**
	2017/2	2022/9	0.14	0.01	*
	2022/10	2023/12	−1.19	0.02	*
Male	2009/1	2016/12	−0.60	<0.01	**
	2017/1	2022/9	−0.01	0.07	
	2022/10	2023/12	−1.15	0.02	*
female	2009/1	2019/12	−0.43	<0.01	**
	2020/1	2020/10	3.24	<0.01	**
	2020/11	2023/12	−0.62	<0.01	**

MPC: estimated Monthly Percent Change by joinpoint regression analysis, * *p* < 0.05, ** *p* < 0.01.

**Table 2:** Summary of temporal fluctuation of SMRs disaggregated by age and sex.

Age	(Males)	Lower Endpoint	Upper Endpoint	MPC	*p*		(Females)	Lower Endpoint	Upper Endpoint	MPC	*p*	
<20		2009/1	2016/3	−0.01	0.93			2009/1	2014/12	−0.45	0.01	*
		2016/4	2023/12	0.35	<0.01	**		2015/1	2023/12	0.07	<0.01	**
								(2020/4)		0.27 (jump)	<0.01	**
20–29		2009/1	2019/11	−0.38	<0.01	**		2009/1	2016/8	−0.65	<0.01	**
		2019/12	2020/9	3.16	0.04	*		2016/9	2019/12	0.41	009	
		2020/10	2023/12	−0.80	<0.01	**		2020/1	2020/11	4.38	0.04	*
								2020/12	2023/12	−0.66	<0.01	**
30–39		2009/1	2016/10	−0.54	<0.01	**		2009/1	2019/12	−0.55	<0.01	**
		2016/11	2021/4	0.22	0.04	*		2020/1	2020/10	4.72	0.02	*
		2021/5	2023/12	−0.48	0.03	*		2020/11	2023/12	−0.83	<0.01	**
40–49		2009/1	2016/11	−0.76	<0.01	**		2009/1	2019/12	−0.47	<0.01	**
		2016/12	2021/12	0.08	0.29			2020/1	2020/10	4.00	0.03	*
		2022/1	2022/3	8.17	0.48			2020/11	2023/12	−0.88	<0.01	**
		2022/4	2023/12	−1.23	<0.01	**						
50–59		2009/1	2013/11	−0.98	<0.01	**		2009/1	2020/4	−0.32	<0.01	**
		2013/12	2022/1	−0.27	<0.01	**		2020/5	2020/8	16.59	<0.01	**
		2022/1	2022/4	9.81	0.56			2020/9	2023/12	−0.47	0.01	*
		2022/5	2023/12	−1.90	<0.01	**						
60–69		2009/1	2016/9	−0.82	<0.01	**		2009/1	2020/2	−0.59	<0.01	**
		2016/10	2022/1	−0.16	0.05	*		2020/2	2020/9	3.02	0.14	
		2022/2	2022/4	11.44	0.44			2020/9	2023/12	−0.64	<0.01	**
		2022/5	2023/12	−1.78	<0.01	**						
70–79		2009/1	2017/2	−0.39	<0.01	**		2009/1	2020/5	−0.43	<0.01	**
		2017/3	2023/12	−0.28	<0.01	**		2020/7	2020/7	9.47	0.79	
								2020/7	2023/12	−0.91	<0.01	**
>80		2009/1	2022/1	−0.30	<0.01	**		2009/1	2020/4	−0.55	<0.01	**
		2022/2	2022/7	4.56	0.55			2020/4	2020/7	5.08	0.83	
		2022/8	2023/12	−1.98	0.01	*		2020/7	2023/12	−0.60	<0.01	**

MPC: estimated Monthly Percent Change by joinpoint regression analysis, * *p* < 0.05, ** *p* < 0.01, (jump): fluctuation of female SMR under 20 years was analyzed using jump model of joinpoint regression analysis (set at the COVID-19 pandemic outbreak in Japan, April 2020).

## Data Availability

All raw data are publicly available to any persons via Japanese national databases from the “Basic Data on Suicide in the Region” (BDSR) in MHLW (https://www.mhlw.go.jp/stf/seisakunitsuite/bunya/0000140901.html) (accessed on 31 January 2024) and “Surveys of Population, Population Change and the Number of Households based on the Basic Resident Registration” in e-Stat (MIAC) (https://www.e-stat.go.jp/en/statistics/00200241) (accessed on 31 January 2024).
